# Platelets and their chemokines in atherosclerosis—clinical applications

**DOI:** 10.3389/fphys.2014.00294

**Published:** 2014-08-08

**Authors:** Philipp von Hundelshausen, Martin M. N. Schmitt

**Affiliations:** ^1^Institute for Cardiovascular Prevention, Ludwig-Maximilians-University of MunichMunich, Germany; ^2^German Centre for Cardiovascular Research (DZHK), Partner Site Munich Heart AllianceMunich, Germany

**Keywords:** atheroscleosis, chemokines, adhesion molecules, platelet aggregation inhibitors, platelet count, heteromers, receptors, cell surface

## Abstract

The concept of platelets as important players in the process of atherogenesis has become increasingly accepted due to accumulating experimental and clinical evidence. Despite the progress in understanding the molecular details of atherosclerosis, particularly by using animal models, the inflammatory and thrombotic roles of activated platelet s especially in the human system remain difficult to dissect, as often only the complications of atherosclerosis, i.e., stroke and myocardial infarction are definable but not the plaque burden. Platelet indices including platelet count and mean platelet volume (MPV) and soluble mediators released by activated platelets are associated with atherosclerosis. The chemokine CXCL4 has multiple atherogenic activities, e.g., altering the differentiation of T cells and macrophages by inhibiting neutrophil and monocyte apoptosis and by increasing the uptake of oxLDL and synergizing with CCL5. CCL5 is released and deposited on endothelium by activated platelets thereby triggering atherogenic monocyte recruitment, which can be attenuated by blocking the corresponding chemokine receptor CCR5. Atheroprotective and plaque stabilizing properties are attributed to CXCL12, which plays an important role in regenerative processes by attracting progenitor cells. Its release from luminal attached platelets accelerates endothelial healing after injury. Platelet surface molecules GPIIb/IIIa, GP1bα, P-selectin, JAM-A and the CD40/CD40L dyade are crucially involved in the interaction with endothelial cells, leukocytes and matrix molecules affecting atherogenesis. Beyond the effects on the arterial inflammatory infiltrate, platelets affect cholesterol metabolism by binding, modifying and endocytosing LDL particles via their scavenger receptors and contribute to the formation of lipid laden macrophages. Current medical therapies for the prevention of atherosclerotic therapies enable the elucidation of mechanisms linking platelets to inflammation and atherosclerosis.

## Role of platelets in atherogenesis

Despite the increasing knowledge about the intricate pathogenesis of atherosclerosis our therapeutic achievements have not much further evolved since the approval of statins. Various cell types and numerous mediators have been identified to contribute in exacerbating or resolving atherosclerotic lesions and are thus principally to be considered as potential targets (Weber and Noels, 2011). The relevance of platelets for atherogenesis, at least for the human system, remains controversial and incompletely understood.

## Platelet indices

One of the early studies giving rise to the hypothesis of platelets as atherogenic factor came from a prospective trial that measured the platelet count and ADP responsiveness in ~500 healthy middle aged men which were followed up over 13 years. Patients presenting with the highest quartile of circulating platelets had an increased risk of coronary death, whereas non-fatal coronary events were not associated. Additionally, patients with a fast and short platelet response to ADP were at higher risk than patients with a slow response to ADP (Thaulow et al., 1991). Of the classical Framingham risk factors only smoking was positively correlated with platelet count and adjustment for smoking still revealed an association of the platelet count and fatal coronary heart disease. As atherosclerotic lesion development was not monitored, the results can be interpreted in a way that highly reactive and numerous platelets represent a risk for a non-resolving platelet thrombus and/or that reactive platelets lead to an increase in vulnerable plaques. In the ARIC trial (Atherosclerosis Risk in Communities) however, ~15,000 coronary healthy patients were followed over 5 years and a high platelet count was suggestive but not significantly associated with coronary disease incidence including non-fatal events (Folsom et al., 1997). Other platelet indices such as mean platelet volume (MPV) have been reported to represent a measure for platelet activation. A meta-analysis comprising 24 trials and a total of ~6000 patients found MPV to be a cardiovascular risk factor associated with acute myocardial infarction (AMI), mortality following myocardial infarction, and restenosis following coronary angioplasty (Chu et al., 2010). MPV has been reported to be increased in hypertension, dyslipidemia, and inflammation and may be reduced by statins (Nadar et al., 2004; Coban and Afacan, 2008).

Lifestyle and nutrition are important factors affecting atherogenesis and are amenable to behavioral changes improving the individual prognosis. For example high intake of saturated fat is believed to be atherogenic, as it correlates positively in most countries with high cardiovascular mortality. In France the situation seems to be paradoxical as cardiovascular mortality is comparatively low despite a high consumption of saturated fats and equal distribution of traditional risk factors. Platelet inhibition by alcohol and unspecified ingredients of wine have been a potential explanation (Renaud and Delorgeril, 1992; Renaud et al., 1992; Rimm et al., 1999). Resveratrol is such a prominent candidate and has been promoted to prevent cardiovascular mortality, but lately failed to hold promises (Semba et al., 2014). As mentioned above, the number of circulating platelets is associated with cardiovascular events. The type of diet not only influences cholesterol levels but as well the platelet count. In the Moli-Sany study ~15,000 healthy Italians were stratified according to the type of diet they adhered. A mediterranean diet correlated with a low platelet count (Bonaccio et al., 2014). The perception of the platelet count as therapeutic target was picked up by an experimental approach in baboons reducing thrombopoiesis by blocking thrombopoietin using antiserum which reduced platelets by 40% and inhibited thrombosis but not hemostasis (Tucker et al., 2010).

## Chemokines and soluble immune mediators

Platelet activation results in the release of soluble immune modulators that are stored in α or dense granules or, in the case of IL-1β, are processed in the cytoplasm. Platelet degranulation determined by CD63 upregulation correlated with the progression of the carotid intima-media-thickness (Fateh-Moghadam et al., 2005).

### Dense granules

Dense granules are packed with small molecules such as pyrophosphates and nucleotides (Mcnicol and Israels, 1999). Moreover, proteomics revealed more than 40 proteins including 14-3-3zeta which can be released by activated platelets and was detectable in human atherosclerotic lesions but not normal aortic tissue (Hernandez-Ruiz et al., 2007). Dense granule release contributes crucially to atherothrombosis and atherosclerosis like remodeling, as HSP3^−/−^ deficient mice that have a defect in dense granule secretion developed less atherosclerosis (King et al., 2009).

### Alpha-granules

Platelet mediators affecting inflammation such as chemokines and growth factors (TGFβ) are kept in α-granules. Some chemokines such as CXCL4 and CXCL7, are amongst the highest expressed proteins in platelets and are detectable in other cell types only in low amounts under physiologic conditions (Karshovska et al., 2013).

#### CXCL7

The role of CXCL7 in atherosclerosis is not well-understood. From the gene CXCL7 the proteins platelet basic protein, beta-thromboglobulin and CTAPIII arise by proteolytic cleavage and are not chemotactic. Only after their release and further N-terminal shortening they bind the receptors CXCR1 and CXCR2, thereby prompting neutrophils and endothelial progenitor cells to migrate (Gleissner, 2012b).

#### CXCL4

CXCL4 was the first of around 50 members of the chemokine family to be cloned and discovered in releasates from platelets (Von Hundelshausen et al., 2007). Physiologic plasma levels of CXCL4 are much higher than of other chemokines. CXCL4 inhibits the proliferation, apoptosis and primes the differentiation of cell types both of the adaptive and innate immune system at high (micromolar) concentrations, which disfavors signaling via G protein coupled receptors. In keeping with this, although the chemokine receptor CXCR3 binds CXCL4, not all of CXCL4-dependent effects are explicable by CXCR3 signaling. Therefore, the principle how CXCL4 exerts its effects remains largely obscure.

In humans, CXCL4 was detected in early and late atherosclerotic lesions of the carotid artery, correlating with the histological and clinical severity of the disease (Pitsilos et al., 2003). CXCL4 deficiency in wildtype and apoE^−/−^ mice and the transplantation of CXCL4 deficient bone marrow into apoE^−/−^ mice on diet results in smaller atherosclerotic lesions with reduced macrophage infiltration (Sachais et al., 2007; Koenen et al., 2009). In addition, CXCL4 plays a role in T cell-platelet interactions, which contribute to the pathogenesis of atherosclerosis (Li, 2013). Co-culture of human platelets with activated CD4(+) T cells resulted in an increased secretion of IFN-γ by soluble mediators including CXCL4 and CCL5 as well as direct cell–cell contacts and skewing toward TH_1_, TH_17_, and regulatory T cell phenotypes (Gerdes et al., 2011). CXCL4 is a potent inhibitor of proliferation for various cell types including T cells, but surprisingly stimulated selectively the proliferation of regulatory T cells (Liu et al., 2005). Moreover, CXCL4 promotes neutrophil and monocyte survival by limiting apoptosis (Scheuerer et al., 2000; Hartwig et al., 2014) and at the same time alters the monocyte phenotype into a subtype that neither represents a classical macrophage nor dendritic cell (Fricke et al., 2004). These findings were corroborated in the sense that in the absence of M-CSF, CXCL4-induced macrophages display a distinct transcriptome and this type of macrophages was suggested to be termed M4 and associate with atherosclerosis (Gleissner et al., 2010). CD163 was one of the most prominently downregulated genes by CXCL4 and both genes correlated inversely in human atherosclerotic plaque material (Gleissner et al., 2010; Gleissner, 2012a). CD163 is a scavenger receptor for haptoglobin-hemoglobin complexes (Hp-Hb) which plays an important role in the clearance of hemoglobin, thereby upregulating heme oxygenase-1 (HMOX1) activity. CD163 deficient macrophages did not upregulate HMOX-1 after challenge with Hp-Hb. Hemoglobin occurs in advanced atherosclerosis and plaque hemorrhage driving a novel protective macrophage subset (Mhem), which seems to be the natural counterpart of the M4 type. In line with these results and the role of iron metabolism in atherosclerosis, macrophages that display high levels of CD163 and contain intracellular iron are considered atheroprotective (Boyle et al., 2012; Hopkins, 2013). Controversially, the conditional deletion of HMOX-1 in murine macrophages protected mice from obesity-induced inflammation and insulin resistance suggesting an atheroprotective role of HMOX-1 (Jais et al., 2014).

An additional mechanism how CXCL4 might influence atherogenesis is an enhanced recruitment of lipids to arteries and macrophages. Already very early studies pointed toward a role of phagocytosed platelets to the fatty alterations of monocytes (Chandler and Hand, 1961). More recent experiments found that oxLDL particles are bound by CXCL4, resulting in enhanced uptake by macrophages finally leading to foam cell formation (Nassar et al., 2003).

Lastly, and more speculative as the role of IL-17 in atherosclerosis is controversially discussed, CXCL4 might influence atherosclerosis by limiting Th17-dependent inflammatory processes as CXCL4 was shown to limit Th17 differentiation (Shi et al., 2014).

#### CCL5

CCL5 is among the highest expressed chemokines on transcript and protein level in platelets (Karshovska et al., 2013) and activates mainly CCR5 and CCR1 receptors. Contact of circulating platelets, their microparticles or releasates with endothelium leads to a deposition of CCL5 and other mediators to the endothelial GAG-decorated surface or vessel lumen mediating monocyte activation and adhesion (Von Hundelshausen et al., 2001). Blocking CCL5 receptors in the mouse by injecting Met-RANTES reduces atherosclerosis not only by preventing monocyte recruitment to the vessel wall, but as well by limiting the number of circulating inflammatory monocytes (Veillard et al., 2004; Combadiere et al., 2008).

The expression of the ATP-cassette transporter ABCB6 is restricted to megakaryocyte-erythrocyte progenitors. Its genetic deletion accelerates atherosclerosis and is associated with elevated plasma levels of CCL5, an expansion of the platelet count, increase in MPV, and elevation of activation markers such as P-selectin, and leukocyte-platelet complexes (Murphy et al., 2014). A potential therapeutic strategy and proof of principle of an anti-inflammatory atheroprotective therapy could be the evaluation of the CCR5 antagonist maraviroc (UK-427857) that is already in clinical use as HIV entry blocker and which was shown in the murine system to reduce atherosclerosis without affecting lipid levels (Cipriani et al., 2013). As maraviroc is well-tolerable, a further exploration in the prevention of human atherosclerosis is warranted.

#### CXCL4-CCL5 heteromers

We found that CXCL4 increases the propensity of CCL5 to trigger monocyte arrest by synergistic interaction and formation of CXCL4-CCL5 complexes. Additionally, transfectants expressing CCR1 and CCR5 variants demonstrated the importance of CCR1 and its third external loop for the activity of CXCL4-CCL5 heteromers (Von Hundelshausen et al., 2005; Kramp et al., 2013). Modeling of the interface and design of a corresponding peptide disrupting the interaction partners provided evidence that the complexes are atherogenic and that injection of the peptide inhibitor prevents in part atherosclerotic lesion formation (Koenen et al., 2009). A variant of CXCL4 with substitutions of three amino acids in the C-terminus is expressed by human but not murine platelets. This variant has low affinity for heparin and CCL5 and lacks synergistic monocyte adhesion (Sarabi et al., 2011; Karshovska et al., 2013; Kuo et al., 2013). Moreover, injection of activated platelets in atherosclerosis-prone mice resulted in a P-selectin-dependent endothelial deposition of CCL5 and CXCL4 and increased lesion formation (Huo et al., 2003).

#### CXCL12

CXCL12 or stromal cell derived factor 1 (SDF-1α) is a vital and well-studied chemokine, which is expressed by various cell types and stored in platelet α-granules. As key mediator of regenerative processes CXCL12 binds and regulates homeostasis, localization and trafficking of endothelial and smooth muscle progenitor cells via CXCR4 and CXCR7. Using the regenerative properties of CXCL12 has been envisioned to be applicable in myocardial infarction and arterial injury (Liehn et al., 2011; Chatterjee and Gawaz, 2013). Its role in atherosclerosis depends on the specific environment, cell type and pathophysiological setting (Doring et al., 2014).

Genome-wide-association studies discovered a relationship of myocardial infarction with single nucleotide polymorphisms (SNP) on chromosome 10q11 near the CXCL12 gene as a powerful predictor for the susceptibility of coronary atherosclerosis and myocardial infarction in association with higher CXCL12 plasma levels (Myocardial Infarction Genetics et al., 2009; Mehta et al., 2011, 2013). Another study confirmed the same SNP rs501120(T/T) to be associated with increased intima-media-thickness and atherosclerosis but in contrast to Mehta et al. to be associated with lower CXCL12 plasma levels (Kiechl et al., 2010).

The systemic application of CXCL12 by injection into apoE^−/−^ mice mobilizes smooth muscle progenitor cells that enter the vascular wall after partial ligation of the carotid artery, leading to a more stable plaque phenotype (Akhtar et al., 2013). This situation may be mimicked when platelets get activated and release substantial amounts of CXCL12. Moreover, platelets express CXCR4 and CXCR7, which get upregulated in patients with coronary artery disease. Therefore, platelets maybe activated in an autocrine way as CXCL12 is a potent platelet agonist abundantly expressed in atherosclerotic plaques (Abi-Younes et al., 2000; Rath et al., 2014). Moreover, platelets release CXCL12 after stimulation with thrombopoietin and soluble c-kit ligand enhancing neovascularization by mobilization of CXCR4+VEGFR1+ hemangiocytes in a model of hind limb ischemia (Jin et al., 2006). Via the sympathetic nerve system and the adrenoreceptor β3 the systemic effects of ischemia result in a decrease of bone marrow derived CXCL12 and other retention factors. This triggers a subsequent release of circulating progenitor cells that enhance the arterial infiltration of monocytes and exacerbate atherosclerotic lesions, which could explain why human atherosclerosis is accelerated after ischemic events (Dutta et al., 2012). After activation by collagen or the adhesion to endothelial cells, human platelets release and present CXCL12 on their surface. The presented CXCL12 activates CXCR4 on CD34+ progenitor cells, which leads to adhesion and to endothelial differentiation (Stellos et al., 2008). The pathogenesis of neointima formation shares several characteristics to atherogenesis. In the area of arterial injury and endothelial denudation, platelets attach readily to the intimal matrix proteins displaying CXCL12, P-selectin and activated GPIIb/IIIa. This facilitates the subsequent recruitment of bone marrow derived progenitors that aid in the following reparative processes (Massberg et al., 2006).

In principle, with Plerixafor/AMD3100 an approved CXCR4 antagonist is available, which seems to be well-tolerated at least for several months as tested in patients with a gain of function mutation in CXCR4 (WHIM syndrome) (Mcdermott et al., 2014). Blocking CXCR4 inhibits the CXCL12-mediated retention in the bone marrow leading to a release of leukocytes and progenitor cells. This renders CXCR4-antagonists useful to mobilize and collect hematopoietic stem cells before autologous stem cell transplantation. Blocking CXCR4 in murine vascular injury models also reduced neointima formation (Karshovska et al., 2008). In a murine model of atherosclerosis the inhibition of the CXCR4-CXCL12 axis by AMD or transplantation of CXCR4 deficient bone marrow led to an increased number of circulating leukocytes (predominantly neutrophils) correlating with atherosclerotic lesions (Zernecke et al., 2008). Another receptor for CXCL12 is CXCR7, now termed atypical chemokine receptor 3 (ACKR3) because it does not transduce signals via G-proteins (Bachelerie et al., 2014). In humans CXCR7 has at least one more ligand, CXCL11 which is naturally absent in C57/B6 mice. Deletion of ACKR3/CXCR7 in apoE^−/−^ mice results in increased atherosclerotic lesion formation and the application of a small molecule ACK3 agonist decreases atherosclerosis by a mechanism that relates to the expression of ACK3 in white adipose tissue, where it takes part in cholesterol metabolism (Li et al., 2014). How platelet-derived CXCL12 affects specifically atherogenesis has yet to be elucidated, e.g., by using models with a platelet-specific CXCL12 deletion.

#### CXCL16

Together with CX3CL1, CXCL16 is the only chemokine that contains a transmembrane domain which enables cell adhesion. CXCL16 is an inflammation marker, expressed by numerous cell types and is associated with atherosclerosis, and acute coronary syndromes in humans (Lehrke et al., 2007). After cleavage it functions as a chemokine attracting T cells. Attached to the cell membrane it is an adhesion receptor and endocytoses oxidized lipoproteins. A constitutive CXCL16 knockout leading to decreased cholesterol efflux shows enhanced lesion size attributable to protective scavenger receptor properties rather than its chemokine functions (Aslanian and Charo, 2006; Barlic et al., 2009). In contrast, CXCR6, the exclusive CXCL16 receptor, has been described to promote atherosclerosis in a cell unspecific CXCR6 knockout model by enhancing T-cell homing and macrophage accumulation (Galkina et al., 2007). Both CXCR6 and CXCL16 are expressed by platelets and transduce PI3K/Akt signaling leading to platelet activation and adhesion under high shear stress (Borst et al., 2012). CXCL16 is upregulated upon platelet activation and in acute coronary syndrome (Seizer et al., 2011). Cell and platelet-specific models have not been used so far.

#### CXCL5

CXCL5 (LIX/ENA-78) plasma and tissue levels are increased during atherogenesis in apoE^−/−^ mice and CXCL5 seems to be atheroprotective by enhancing the cholesterol efflux capacity of macrophages and regulating foam cell formation (Rousselle et al., 2013). The source of CXCL5 could potentially as well be platelets that express CXCL5 at a high level (Karshovska et al., 2013).

#### MIF

The cytokine macrophage migration inhibitory factor (MIF) is structurally and functionally related to the chemokine family as it resembles the CXCL8-dimer and binds and activates CXC chemokine receptors playing an aggravating role in atherogenesis (Bernhagen et al., 2007; Weber et al., 2008; Tillmann et al., 2013). Platelets are a previously unrecognized source of MIF, which is stored in α-granules but does not co-localize with other chemokines or growth factors including VEGF. This may explain the differential release upon ADP and oxLDL stimulation compared to CXCL12 (Strussmann et al., 2013).

**Figure 1:**
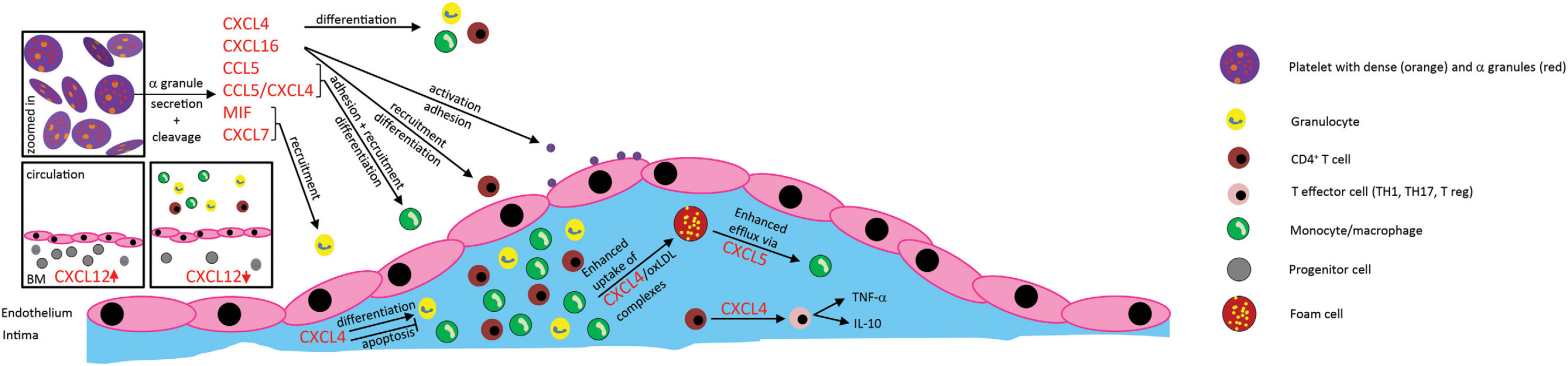
**Effects of platelet-derived chemokines on inflammatory cells and atherogenesis**. Chemokines are stored in α-granules and secreted upon activation. Most platelet-derived chemokines act on the one hand on inflammatory cell differentiation and apoptosis. On the other hand, they act on inflammatory cell adhesion to the endothelium with subsequent transendothelial migration. Thus, platelet-derived chemokines are crucial driving factors for atherogenesis. Further effects of platelet-derived chemokines are the retention of bone-marrow leukocytes and regulation of lipid transport.

The release of chemokines from platelets is linked to platelet activation, but depending on the individual chemokine, atherogenic or protective effects occur (Figure 1). Proteins in α-granules seem to be unequally distributed and numerous α-granule cytokines and growth factors are not co-localized, which argues for the possibility of a selective release. This issue has been addresses by several studies but remains controversial (Italiano et al., 2008; Kamykowski et al., 2011). It has been found that platelet secretion follows a fast, medium, and slow rate and that cargo release might be rather a stochastic process depending on the structure or trafficking of the granule than a specifically targeted process (Jonnalagadda et al., 2012).

#### Plasminogen activator inhibitor-1

PAI-1 excess promotes the development of intravascular thrombosis and atherosclerosis (Vaughan, 2005). Large amounts of active PAI-1 are continuously produced by platelets (Brogren et al., 2004). PAI-1 in mice affects atherosclerosis dependent on the location. In the aortic arch the knockout or overexpression in apoE^−/−^ or LDLR^−/−^ mice did not alter atheroprogression (Sjoland et al., 2000). The same result was reproduced by a different group that additionally investigated atherosclerosis in the carotid artery detecting a protection in PAI-1 knockout mice (Eitzman et al., 2000). Consistent with the difference in atherogenesis dependent from the arterial bed, fibrin deposition was more intense in the carotid artery than in lesions of the aortic arch. PAI-1 levels in humans correlate with IMT in diabetics (Adly et al., 2014).

## Platelet adhesion receptors and co-stimulatory molecules

### GPIIb/IIIa and GP1bα

Activated platelets have been documented to adhere to activated endothelial cells in mouse models via surface expressed adhesion molecules. The integrin GPIIb/IIIa (αIIbβ3) is a central player in platelet adhesion and its absence in mice completely prevents platelet adhesion to the carotid artery of apoE^−/−^ mice *in vivo* and the presence of an active von Willebrand Factor (VWF)-receptor GP1bα is required to a large part, too. Blocking or genetic deficiency of either one resulted in a considerable reduction of atherosclerosis by reduced monocyte recruitment (Massberg et al., 2002, 2005). A mechanistic aspect could be that CXCL4 release by platelets was dependent on functional GPIIb/IIIa *in vitro* (Zokai et al., 2001). Opposite to this finding, the α- and dense granule release reaction of surface adherent platelets was not GPIIb/IIIa dependent as shown with the blocking antibody fragment GPIIb/IIIa abciximab in experiments with isolated human platelets (Ilveskero and Lassila, 2003). These controversial effects of GPIIb/IIIa inhibitors on platelet granule release are possibly due to the fact that mediators activating platelets cause both, aggregation through inside-out signaling of GPIIb/IIIa and secretion via an alternative pathway. In the latter case, outside-in signaling of the integrin is not required leading to the notion that GPIIb/IIIa inhibition results in a dissociation of the aggregatory and secretory response (Tsao et al., 1997; Ogawa et al., 2002; Naimushin and Mazurov, 2003).

Conflicting results exist in addition whether GPIIb/IIIa inhibitors are able to reduce the formation of atherogenic platelet-leukocyte complexes (Klinkhardt et al., 2002).

These experimental conditions are mimicked in patients with inherited platelet disorders such as Glanzmann thrombasthenia (GPIIb/IIIa), Bernard Soulier Syndrome (GPIbα) and von Willebrand disease (VWD). From studies of Glanzmann patients we know that the absence of GPIIb/IIIa does not fully protect from atherosclerosis since ultrasound of the carotid bifurcation revealed plaques in 4 of 7 patients (Shpilberg et al., 2002). Therefore, platelet-vessel wall interactions via GPIIb/IIIa seem not to be required in human atherosclerosis and may be replaced functionally by other platelet receptors. Orally administered GPIIb or GPIIIa inhibitors would be of use to conclude on this question but have been abandoned due to increased mortality and exist only as i.v. drugs. The reason for the increased mortality in phase III trials with oral GPIIb/IIIa antagonists has not been elucidated and pharmacokinetics rather than the mechanism itself may be blamed so that the quest for appropriate antagonists continues (Bledzka et al., 2013). Junctional molecules altering GPIIb/IIIa activity are additional potential therapeutic candidates. For instance, JAM-A (junctional adhesion molecule A), a member of the superimmunoglobulin class of adhesion molecules is expressed by platelets. Surprisingly, JAM-A deficiency leads to an increase of GPIIb/IIIa-mediated outside-in signaling (Naik et al., 2012).

### VWF

Not much is known about the relevance of human VWF and its receptor complex for atherosclerosis. In a rabbit model of atherosclerosis, it was shown that endothelial VWF recruited platelets to atherosclerosis-prone sites in response to hypercholesterolemia (Theilmeier et al., 2002). The complete absence of VWF in humans (VWD type 3) seems not to protect from atherosclerosis as a study with relatively young individuals (average 37 years) suggested. Healthy controls and patients were examined by ultrasound and showed a comparable percentage of plaques and Intima-Media-Thickness (Sramek et al., 2004).

### P-selectin

P-selectin is upregulated on endothelial cells and platelets upon activation and mediates platelet and leukocyte rolling on the endothelium. Activated platelets rapidly release P-selectin by shedding but continue to circulate and function (Michelson et al., 1996). Platelets express the fractalkine (CX3CL1) receptor CX3CR1 and get activated by inflamed endothelial cells through their surface displayed CX3CL1 triggering P-selectin exposure on adherent platelets, which thereby initiates the local accumulation of leukocytes under arterial shear (Schulz et al., 2007). Increased soluble P-selectin levels of apparently healthy women predict future vascular events (Ridker et al., 2001). High levels of P-selectin on platelets are associated with an increased Intima-Media-Thickness (Koyama et al., 2003). In a mouse model of atherosclerosis, investigating the promotion of atherosclerosis by an adoptive transfer of P-selectin positive or negative bone marrow platelets, in addition to endothelial P-selectin, platelet P-selectin contributed to lesion formation (Burger and Wagner, 2003). Platelet P-selectin supports the recruitment of monocytes and other leukocytes by aiding the formation of platelet-leukocyte complexes and facilitating the deposition of inflammatory platelet mediators on endothelial cells (Schober et al., 2002; Huo et al., 2003). A novel oral non-peptide inhibitor of P-selectin failed to decrease the formation of monocyte-platelet aggregates (Japp et al., 2013). A P-selectin blocking monoclonal antibody has been tested in NSTEMI patients, but had only a small but positive effect on myocardial necrosis and is planned to be applied in the chronic setting of atherosclerotic peripheral artery disease (Tardif et al., 2013) (NCT00760565).

### The CD40-CD40L dyade

CD40 ligand (CD154, CD40L) is a transmembrane protein of the TNF-family and one of the best characterized co-stimulatory molecules. CD40L activates CD40 thereby stimulating atherogenic immune responses (Lievens et al., 2009). CD40 and CD40L are expressed by numerous cell types, but in the circulation CD40L is mainly derived as cleavage product from activated platelets (Henn et al., 1998). Whole body deficiency of CD40L or of hematopoietic CD40 reduces atherosclerotic lesion size and induces a stable plaque phenotype through signaling of the intracellular adaptor TRAF6 but not TRAF2/3/5 (Lutgens et al., 1999, 2010). Platelet CD40L interaction with endothelial cells induces the release or upregulation of chemokines (e.g., CCL2 and CCL5), adhesion molecules (e.g., ICAM-1, VCAM-1), metalloproteases (e.g., MMP-1, -2, -3, and -9) and tissue factor supporting platelet-endothelium interaction, but also leukocyte recruitment via the formation of platelet-leukocyte-aggregates (Lievens and Von Hundelshausen, 2011). Atherosclerotic lesion formation was increased if activated wild type platelets were injected into apoE^−/−^ mice compared to CD40L-deficient platelets. The responsible mechanisms comprised less platelet leukocyte aggregates and no depletion of T-regs by CD40L deficient platelets (Lievens et al., 2010). Vice versa, CD40L-positive T cells activate platelets through a CD40-dependent pathway resulting in CCL5 release and T cell recruitment (Danese et al., 2004). Furthermore, ligation of platelet CD40 with a recombinant soluble CD40L augments P-selectin expression, α-granule and dense granule release and the typical shape change that is associated with platelet activation. Some atherogenic effects of CD40L are not mediated by CD40 but are explained by the interaction with the I-domain of the integrin MAC-1 that can be blocked by peptide inhibitors (Wolf et al., 2011). Anti-CD40L treatment in patients with systemic lupus erythematodes led to an increase of thrombotic events that are assumed to depend on the Fc part of the antibody, which activates platelets via their Fc-receptor gamma. This could be principally overcome by engineering an inert Fc-part (Sidiropoulos and Boumpas, 2004; Xie et al., 2014). However, the desired immunosuppressive effects of a long-term inhibition are not acceptable in cardiovascular prevention. A resort can be the mentioned selective antagonism of TRAF6 that blocks only the atherogenic pathway of CD40L-CD40 signaling being enabled by the distinct binding site to TRAF1/2/3/5. Controversially, platelet CD40L might have as well anti-inflammatory properties related to the interaction between CD40L and CD40, and exert a hitherto undescribed immunoregulatory action by enhancing IL-10 production and inhibiting TNF-α production by monocytes (Gudbrandsdottir et al., 2013).

## Platelet-derived microparticles in atherosclerosis

Platelet-derived microparticles (PMP) are small phospholipid-vesicles loaded with bioactive substances being shed from aged or activated platelets (Heemskerk et al., 1997). There is growing evidence for PMP playing important roles in atherosclerosis (Tan and Lip, 2005). In healthy subjects, low numbers of PMPs circulating in the blood exert only minor effects like phospatidylserine catalyzed generation of negligible amounts of thrombin (Rautou et al., 2011), but upon activation PMP abundance in the blood rises, as suggested by *ex vivo* activation with TRAP and ADP of platelets from convalescent stroke patients (Lukasik et al., 2013). PMPs were identified as transcellular delivery systems for chemokines such as CCL5 promoting monocyte recruitment and atherosclerosis (Mause et al., 2005). Moreover, PMPs were shown to interact with monocytic MM6 cells subsequently inducing integrin (α5), interleukin (IL-1β, -7, -11) and CCL5 expression (Setzer et al., 2006). Besides augmented monocyte recruitment, enhanced adherence of murine progenitor cells to sites of wire-induced arterial injury had been demonstrated to be mediated by PMPs pointing toward an additional regenerative role for PMP (Mause et al., 2010). Taken together, these data provide strong evidence for a contribution of PMPs in atherosclerotic lesion formation.

## Role of platelets in cholesterol accumulation

A detailed review covering the major aspects of the interplay of platelets with native and modified lipoproteins summarizing the contribution of platelets to foam cell formation in atherosclerosis has been published elsewhere (Siegel-Axel et al., 2008).

Platelets bind, modify and endocytose LDL particles as an early *in vitro* study demonstrated by adding activated platelets to smooth muscle cells or macrophages. This induces the formation of intracellular cholesterol ester droplets independent on extracellular lipids and intracellular cholesterol synthesis, which were not observed with resting platelets (Kruth, 1985). Platelets express several receptors for lipoproteins: CD36 (SR-BIII), SR-BI, SR-BII, LOX-1, apoE Receptor 2 and CXCL16 that contribute in these processes. In line with the atherogenic role of blood-borne LDL and the protective role of HDL, LDL, and oxLDL proteins activate platelets in contrast to HDL, which through binding to SR-BI exerts an indirect influence on platelet reactivity via maintaining normal plasma cholesterol homeostasis and generates an inhibitory signal for platelet activation (Korporaal et al., 2011; Nofer and Van Eck, 2011). Lowering LDL cholesterol by statins or lipid apharesis led to a decrease in the MPV (Blaha et al., 2013; Sivri et al., 2013). Stimulation of platelets with oxLDL resulted in the formation of platelet-monocyte-aggregates (PMA) and phagocytosis of platelets in whole blood and increased oxLDL uptake by monocytes, which was dependent on platelet CD36 and the release of CXCL4 (Badrnya et al., 2014). Interestingly, the same study reported that platelet inhibition by aspirin or clopidogrel was effective in preventing oxLDL uptake and PMA formation, attributing cardiovascular beneficial effects of aspirin and clopidogrel to these mechanisms.

Hypercholesterolemia increases the number of circulating neutrophils and monocytes but as well the platelet count by enhanced production via the cholesterol-efflux transporter ABCG4 on megakaryocyte progenitors, which in turn affects cholesterol-sensing LYN- kinase and signaling of the thrombopoietin receptor c-mpl (Murphy et al., 2013).

## Mechanisms of platelet-mediated atherogenesis get apparent by cardiovascular drugs

Linking inflammation and thrombosis supports the hypothesis that agents with both anti-inflammatory and antiplatelet effects may reduce vascular inflammation and limit acute and long-term thrombotic events.

## P2Y12R antagonists

Inflammation, cell death and activation lead to the release of nucleotides such as ATP and ADP by various cell types including platelets binding and activating purinergic receptors. Platelets express the ADP receptors P2Y12 (Gi linked) and P2Y1 associating with Gq as well as the ATP activated ion channel P2X1. P2Y1 signaling activates phospholipase C, which will be followed by platelet shape change, whereas activation of the P2Y12R triggers platelet aggregation via GPIIb/IIIa and is the target of well-established antagonists such as clopidogrel, prasugrel, and ticagrelor that are applied in the prevention of arterial thrombosis (Idzko et al., 2014). Given the importance of nucleotide signaling in inflammation and the concept of platelets being inflammatory cells with immunologic tasks, P2Y12R should play a role in platelet-mediated inflammation and atherosclerosis.

Indeed, P2Y12R deficient mice are partially protected from atherosclerosis by a reduction of α-granule release leading to a lower P-selectin expression and lower plasma levels of CXCL4 (Li et al., 2012). Similar to diet-induced atherosclerosis, transplant-associated atherosclerosis features arterial leukocyte infiltration. In P2Y12R deficient mice platelets expressed lower levels of CD40L and formed fewer aggregates with leukocytes additional to mediating lower adhesion molecule levels in endothelial cells (Yashiro et al., 2009). The use of P2Y12R antagonists in animals brought conflicting results. Ticlopidine, a first generation P2Y12 inhibitor, and clopidogrel showed atheroprotective effects (Jawien et al., 2007; Afek et al., 2009), which could not be reproduced in another study using clopidogrel, aspirin or a combination thereof (Schulz et al., 2008).

Adding clopidogrel to aspirin in the CHARISMA trial in stable CAD patients did not improve outcome or reduce cardiovascular events over 2 years thus failing to translate the positive results of the CAPRIE trial into primary prevention that were obtained in the setting of AMI (Chen et al., 2005; Bhatt et al., 2006). The reasons for the failure of clopidogrel to reduce atherosclerosis in humans despite the positive preclinical data are unclear but without understanding the cause it will be difficult to encourage additional clinical trials addressing the clinical efficacy of other P2Y12R antagonists (such as prasugrel or ticagrelor) for the treatment or prevention of chronic inflammation and its complications. Whether long-term clopidogrel treatment (12 months) had an influence on inflammation and whether the effects were stable over time was investigated in the ELAPSE trial (Saw et al., 2008). During 12 months follow-up the C-reactive protein levels of 26 CAD patients remained constant while IL-18 levels increased significantly although platelet function testing did not show a decline in inhibition of aggregation through clopidogrel. After 1 year the surface expression of P-selectin and CD40L, but not activated GPIIb/IIIa was elevated, leading to the conclusion that clopidogrel and maybe in general P2Y12 inhibitors, although still blocking aggregation, could lead to platelet activation and have general inflammatory side effects. It is expected that effective platelet inhibition would most likely lead to lesser chemokine secretion and adhesion molecule expression as exemplarily shown in plasma of patients with type 2 diabetes mellitus, where CCL5- and P-selectin-levels are decreased (Harding et al., 2006).

## Thrombin and thrombin receptor antagonists

A role of the serine protease thrombin for the initiation of atherosclerosis is testified by the presence of thrombin-generating activity in early atherosclerotic lesions and increased atherosclerosis in mice (Iwaki et al., 2006; Borissoff et al., 2011). Thrombin generated at sites of vascular inflammation activates major atheroma-associated cells including endothelial cells, platelets, smooth muscle cells, monocytes, and macrophages producing a wealth of inflammatory mediators and procoagulant activity resulting in a positive feedback loop (Croce and Libby, 2007). Multiple substrates of thrombin binding to exosite I or II are relevant for its biological activity including the activation of proteinase activated receptors 1 and 4, binding to GPIbα as well as the cleavage of several coagulation factors (fibrinogen, XI, V, VIII), which make thrombin an important player linking thrombosis and innate immunity (Lane et al., 2005; Engelmann and Massberg, 2013). Importantly, low concentrations of thrombin are thought to have antithrombotic and inflammatory properties as low concentrations of thrombin are complexed on endothelial cells with protein C, thrombomodulin and the endothelial protein C receptor (EPCR) generating activated protein C thus attenuating the effects of thrombin on coagulation by cleaving FV and VIII as well as transducing anti-inflammatory signals via endothelial PAR-1 (Riewald et al., 2002; Kalz et al., 2014). At higher concentrations thrombin specifically activates platelets through human PAR-1 and PAR-4 corresponding to mouse PAR-4 and PAR-3, respectively, whereas murine PAR-1 is not relevant for platelet activation (Kahn et al., 1998; Major et al., 2003). Human PAR-1 is the high affinity thrombin receptor resulting in a fast and short activation, whereas PAR-4 has a low apparent affinity, but due to its prolonged activation it dominates the signaling over time despite its slow activation rate (Covic et al., 2000).

### Direct thrombin inhibitors

The repertoire of oral anticoagulants has been extended by Factor Xa antagonists and the direct thrombin inhibitor dabigatran which reduces atherogenesis in mouse models. Heterozygeous prothrombin deficient mice or blocking thrombin with dabigatran reduces the size of atherosclerotic lesions in mice and at the same time increases plaque stability through effects on MMPs, endothelial dysfunction and neutrophil recruitment (Kadoglou et al., 2012; Borissoff et al., 2013; Pingel et al., 2014).

These models provide the insight that targeting thrombin activity may be beneficial, but due to the multifaceted effects of thrombin affecting multiple cell types and plasmatic proteins the answer which cell type contributes most to thrombin-dependent atherogenesis remains open. In the setting of secondary prophylaxis where aspirin and often clopidogrel are co-administered caution is needed before adding another therapy affecting hemostasis.

### Thrombin receptor antagonists

The same holds true for the inhibition of thrombin receptors. Here, tissue specific animal models are feasible. Presently, four members of the PAR family have been cloned and identified. Human platelets express and are activated via PAR-1 and PAR-4 to release substantial amounts of cytokines and chemokines which modulate atherosclerosis (see above). The development of PAR knockout mice has provided the unique opportunity to identify and characterize the members of this family of GPCRs, to evaluate the interaction of PARs jointly expressed in common cells and tissues, and better understand the role of PARs in thrombosis, restenosis, vascular remodeling, angiogenesis, and inflammation (Major et al., 2003).

Surprisingly, the deficiency in the major murine platelet thrombin receptor PAR-4 seems not to be atheroprotective in apoE^−/−^ mice, at least at the early stages at 5 and 10 weeks on Western diet, although platelet activation by thrombin was abolished (Hamilton et al., 2009). As PAR-4 is expressed by various other cell types, a compensating effect might be the explanation or relates to the finding that PAR signaling is important for platelet activation and clot formation, but not for the formation of the initial platelet monolayer at sites of injury (Vandendries et al., 2007; Angiolillo et al., 2010). PAR-1 antagonists have therefore been proposed to tackle platelet-mediated thrombosis rather than platelet hemostasis (Angiolillo et al., 2010).

The two selective and reversible PAR-1 antagonists in most advanced clinical development are vorapaxar and atopaxar. The latter has been studied in the phase II LANCELOT trials. The LANCELOT-ACS trial compared placebo and different doses of atopaxar on top of the standard therapy with aspirin and clopidogrel in 603 patients presenting with non-ST-elevation ACS, but failed to demonstrate a difference in hard endpoints.

While ischemic events, as determined by the surrogate parameter continuous ECG monitoring, significantly favored atopaxar, neither hard endpoints nor the incidence of bleedings differed significantly. The treatment of CAD patients with atoxapar did not result in a reduction of inflammatory mediators, at best soluble CD40L decreased (O'donoghue et al., 2012). Another selective PAR-1 antagonist, vorapaxar, has been shown to be safe and well-tolerated in phase I and II studies but was terminated early in a phase III trial treating ACS patients (TRACER) because of increased bleeding compared to not-significantly lower cardiovascular events (Tricoci et al., 2012). In secondary prevention (TRA 2P-TIMI 50) vorapaxar proved to be effective (Morrow et al., 2012) but this benefit was abolished by moderate to severe bleeding, including intracranial hemorrhage.

The concept of thrombin receptors as targets to treat and prevent atherosclerotic complications has partially proven effective but could not solve the dilemma of anti-platelet therapies. However, the receptor PAR-4 could be a potential candidate.

### Heparin

Unfractionated heparin, a family member of the glycosaminoglycans, has been used for decades as a standard anticoagulant in cardiovascular diseases inhibiting thrombin by binding to and increasing the effectivity of antithrombin III (AT). Beyond anticoagulation, heparin and its variants play a role in atherosclerosis, preferably adhering at the endothelial cell lining and inhibiting platelet endothelial cell interactions, preventing lipid uptake, and increasing lipoprotein-lipase A2 activity (Engelberg, 1980). As conventional heparin has to be applied parenterally and longer courses are regularly complicated by CXCL4-heparin complexes provoking heparin-induced thrombocytopenia (HIT), only short term applications are practical and seem to contradict a realistic perspective of heparin as treatment option for the chronic disease atherosclerosis. However, short term heparin application may alter the prognosis during acute ischemia, which is an important driver of atherosclerosis by enhancing the pool and number of circulating monocytes and progenitor cells via CXCL12 (Dutta et al., 2012). It is known that administration of heparin results in a moderate increase in the white blood cell count. Many activities of heparin, mainly by binding to growth-factors and chemokines may be important to understand completely as they mediate the mobilization and recruitment of cells affecting atherosclerosis (Xu and Dai, 2010). Heparin-induced leukocytosis affects less than 1% of patients, requires 6-O-sulfation and is caused by blockade of selectin- and CXCL12-mediated leukocyte trafficking in mice (Zhang et al., 2012). Heparin oligosaccharides inhibit CXCL12 by orthogonally binding to the dimerization interface, promoting oligomerization, and competing with CXCR4, which prevents progenitor cell recruitment (Ziarek et al., 2013).

Patients with atherosclerosis have low intraplatelet stores of CXCL4, but large extracellular deposits reflecting chronic platelet activation and release. Administration of heparin in these patients mobilizes larger amounts of CXCL4 into the circulation than in healthy individuals (O'brien et al., 1984). In terms of antithrombotic activity heparin neutralizes CXCL4 and possibly other activities of the chemokine (Eslin et al., 2004).

The heparin binding motif in CCL5 in the 40s loop is important for its biological activity and contributes to atherosclerosis. Injecting a CCL5 mutant [^44^AANA^47^]-RANTES in LDLR-deficient mice protects from atherosclerosis (Braunersreuther et al., 2008). Designing heparin oligosaccharides may therefore help to selectively modulate chemokine activity (De Paz et al., 2007).

Summing up, heparin might exert beneficial effects on atherosclerotic lesion formation by blocking CXCL12 and CXCL4, but the induction of atherogenic leukocytosis might counter-balance these positive effects and additionally impede healing processes after myocardial infarction.

## Cyclooxygenase-1 inhibitors

Aspirin (acetylsalicyl acid, ASA) is the standard drug in the secondary prevention of myocardial infarction and stroke. It acts in the usually applied low doses as an irreversible inhibitor of cyclooxygenase (COX-1), an enzyme required for the synthesis of prostaglandins such as thromboxane A2, which mediates platelet thromboxane receptor amplifying platelet aggregation (Coccheri, 2010).

Other NSAIDs (Non-Steroidal Anti-Inflammatory Drugs) failed to show a protective effect on myocardial infarction, which might be due to inhibiting cyclooxygenase reversibly in contrast to the irreversible binding of aspirin (Ray et al., 2002). Numerous large-scale clinical trials and meta-analyses have consistently demonstrated the benefit of low doses of aspirin as a secondary prevention measure for recurrent ischemic events in patients with various manifestations of atherothrombotic disease, whereas the effect in primary prevention is small and doubtful (Baigent et al., 2009). In the largest previous placebo-controlled trial of antiplatelet therapy for secondary prevention in patients with stable atherothrombosis or at high risk for vascular disease, clopidogrel plus aspirin was no better than aspirin alone in the overall cohort (Bhatt et al., 2006).

In contrast, experimental studies demonstrated that aspirin is atheroprotective (Paul et al., 2000; Cyrus et al., 2002; Tous et al., 2004). The exposition of isolated human platelets to various platelet agonists results in a differential profile of released platelet proteins, attenuated by aspirin irrespective of which agonist was used (Coppinger et al., 2007). Additionally, both clopidogrel and aspirin were shown to decrease atherosclerosis in rabbits and at the same time reducing P-selectin and thus platelet adhesiveness, as well as MCP-1 expression (Li et al., 2007b).

The lack in efficacy of aspirin and clopidogrel in primary prevention is in striking contrast to the *in vitro* and *in vivo* studies describing an atherogenic role of COX-1-mediated platelet activation leading to an enhanced release of inflammatory mediators and cellular recruitment and the development and progression of atherosclerotic lesions in hyperlipidemic mice and rabbits.

## Statins

**Figure 2:**
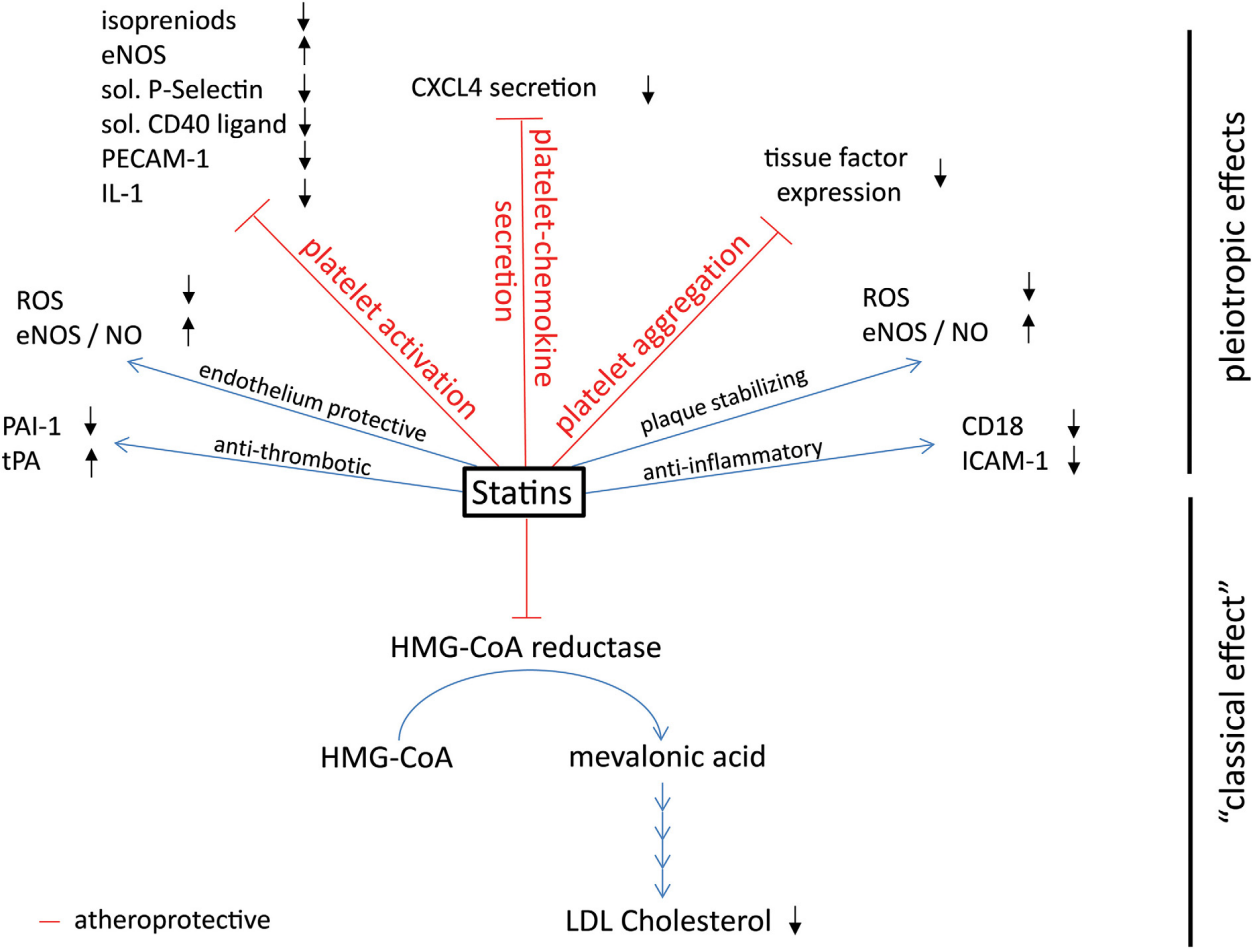
**Effects of platelet-derived chemokines on inflammatory cells and atherogenesis**. Chemokines are stored in α-granules and secreted upon activation. Most platelet-derived chemokines act on the one hand on inflammatory cell differentiation and apoptosis. On the other hand, they act on inflammatory cell adhesion to the endothelium with subsequent transendothelial migration. Thus, platelet-derived chemokines are crucial driving factors for atherogenesis. Further effects of platelet-derived chemokines are the retention of bone-marrow leukocytes and regulation of lipid transport.

Research into fungi and cholesterol led to the development of statins (Endo et al., 1976), which inhibit the 3-hydroxy-3-methyl-glutaryl-CoA reductase (HMG-CoA reductase) which is the first and key enzyme of cholesterol biosynthesis (Figure 2: statins: mechanisms of action), thereby preventing the generation of cholesterol precursors in the liver and reducing LDL-cholesterol, the pivotal cardiovascular risk factor. Cholesterol lowering statins are one of the most commonly used classes of atheroprotective drugs and so far exclusively proven to be beneficial in the primary prevention of myocardial infarction and stroke (Robinson, 2013). In clinical trials some of the protective properties of statins were not correlated with the reduction in cholesterol levels. At present it is unclear whether and to what extent LDL reduction or other modes of actions are responsible for the improved cardiovascular outcome in high risk patients. Anti-thrombotic, anti-inflammatory, endothelium protective, and plaque stabilizing properties are additional attributes of statins and outline the so-called pleiotropic, cholesterol independent, effects (Lefer et al., 2001; Liao and Laufs, 2005; Violi et al., 2013).

The clinical relevance of the antithrombotic effects of statins remains controversial. High-dose atorvastatin in the SPARCL trial (Stroke Prevention by Aggressive Reduction in Cholesterol Levels), decreased the overall rate of recurrent ischemic events in patients with stroke and were overall protective at the expense of increased intracranial hemorrhage (Amarenco et al., 2006). Later, smaller observational cohorts and a meta-analysis however were not confirmative (Biffi et al., 2011). Scientifically, it will be difficult to clearly separate the off- and on-target effects of statins. Moreover, the issue of antithrombotic effects of statins has not been addressed directly by randomized trials.

Nevertheless, antithrombotic pleiotropic properties may be inferred from acute clinical effects when statins not yet have reduced plasma cholesterol. A meta-analysis summarizing the data of 13 studies testing short term, high-dose statins, started just before PCI, demonstrated both a large relative reduction of 44% of periprocedural myocardial infarction irrespective of the clinical setting (ADP antagonist, acute event, etc.) and later a reduction in adverse cardiac events. Due to the short time frame a lipid lowering effect was not yet effective, implying pleiotropic effects (Patti et al., 2011). The PRISM trial (Platelet Receptor Inhibition in Ischemic Syndrome Management) demonstrated a better outcome of ACS patients when they were on statins in the first month after the event, which is unlikely to be caused by remodeling processes of the plaque in such a short timeframe and are rather to be explained by reduced platelet activation and/or attenuated coagulation system. It was observed that statins have an early inhibitory effect on platelets of patients with AMI showing reduced aggregation after challenge with collagen and a markedly lower platelet surface adhesion *in vitro* (Matetzky et al., 2011).

Statins may exert effects on atherosclerotic lesion formation via decreasing long-term platelet activation and platelet-chemokine secretion by various mechanisms. Platelets release nitric oxide (NO) upon activation preventing in a negative feedback loop further platelet activation and recruitment (Freedman et al., 1997). NO is generated in platelets by eNOS (endothelial type III nitric oxygen synthase) utilizing arginine as substrate. Statin treatment leads to an upregulation of eNOS, decreases platelet activation, lowers plasma levels of the platelet chemokines CXCL4 and CXCL7 *in vivo* and protects from cerebral ischemia in normocholesterolemic mice (Laufs et al., 2000). On the other hand, O_2_ radicals will deplete NO. Atorvastatin decreases acutely and simultaneously oxidative stress and platelet activation by directly inhibiting platelet Nox2 (NADPH oxidase) and ultimately platelet isoprostanes and thromboxane (Pignatelli et al., 2012). Therefore, statins may reduce oxidized LDL by lowering LDL and oxidative stress increasing NO availability at the same time. The molecular details, i.e., where exactly statins bind to the enzymes are not yet determined.

Beyond eNOS-dependent effects of statins, animal studies demonstrated additional mechanisms such as inhibiting signaling of the thrombin receptor PAR-4 (Ni et al., 2012). Small GTPases such as Rho, Ras, and Rac play a central role in the signaling and cytoskeletal rearrangements required for platelet activation (Aslan and Mccarty, 2013). RhoA activates GTP-bound ROCK (Rho-associated coiled-coil containing protein kinase), which in turn activates MLC (myosin light chain) and is necessary for the contraction of actin fibers. The intracellular location of Rho-GTPases is dependent on isoprenoid intermediates. Hence, the reduced activity of ROCK in patients with high dose simvastatin is biologically plausible (Liu et al., 2009). Despite the implications of statins as anti-platelet agents, their role for the signaling of small GTPases and the above mentioned mechanisms still need further clarification. The supposedly general effect on small GTPases might be responsible for the statin-mediated blockade of junctional adhesion molecule A (JAM-A) re-localization on endothelial cells under atherogenic conditions, including low shear stress and hyperlipidemia. Under resting conditions, JAM-A is evenly distributed at endothelial cell-cell interfaces, whereas it appears to be redistributed profusely under atherogenic conditions. The junctional localization of JAM-A could be restored upon challenging with a statin likely facilitated by reducing actin stress-fiber formation (Schmitt et al., 2014). Furthermore, high-dose (>5 μM) atorvastatin redistributed proteins, including chemokines, intracellularly to CD63 positive platelet and endothelial multivesicular bodies which was associated with a lower secretion (Hol et al., 2012). The presentation of chemokines on endothelial cells and endothelial JAM-A re-localization through exposure with oxidized low density lipoprotein (LDL) or cytokines was an important prerequisite for the transendothelial migration of inflammatory leukocytes (Jaczewska et al., 2014). Therefore, reducing hot spots of apical JAM-A and chemokine accumulation for migration may mitigate leukocyte infiltration.

Using an atherosclerosis model in rabbits, Li and colleagues furthermore found an association between the reduction in atherosclerotic lesions induced by statins as well as by aspirin or clopidogrel and the reduction of soluble P-selectin. Interestingly, atorvastatin showed a comparable reduction in plasma sP-selectin to clopidogrel treated animals which might be a platelet-dependent effect (Li et al., 2007a). P-selectin on platelets was diminished by 20% after 8 weeks when fluvastatin (40 mg) was compared with placebo (Huhle et al., 1999). Well-correlating levels of sP-selectin and IL-1β in 50 patients at baseline and a comparable decline after 8 weeks of treatment with either simvastatin (20 mg) or aspirin (100 mg) brought the perception that platelet activation (sP-selectin) in hypercholesterolemia correlates with inflammation (IL-1β) and that both, simvastatin and aspirin lower platelet activation to the same extent but surprisingly only simvastatin lowered the inflammation marker CRP. As baseline was compared with solely 8 weeks of treatment, the differentiation in cholesterol-dependent and -independent effects was not feasible (Ferroni et al., 2003).

Studies on patients with hypercholesterolemia demonstrated on the other hand a statin-mediated decrease in platelet expressed and plasmatic soluble CD40 ligand that correlated with cholesterol levels, arguing against an exclusive pleiotropic mechanism and favoring the concept that high concentrations of cholesterol, lipoproteins and its oxidized modifications (oxLDL, mmLDL) activate platelets (Cipollone et al., 2002; Semb et al., 2003; Sanguigni et al., 2005; Undas et al., 2005).

A further, recently discovered molecular mechanism of statin-mediated pleiotropic platelet inhibition regards the platelet molecule PECAM-1. Fluvastatin and simvastatin were found to reduce acute platelet aggregation, dense granule release and thrombus growth *in vitro* and in animal models by activating the signaling of PECAM-1, an inhibitory platelet membrane protein. Adding these statins to platelets reduced collagen-induced platelet activation, stimulated the phosphorylation of PECAM-1, which increased the activity of the phosphatase SHP-2, and subsequently diminished PI3K and AKT signaling (Moraes et al., 2013). However, the immediate mechanism leading to PECAM-1 phosphorylation remained still concealed. An important indirect AKT inhibitor is the phosphatase PTEN (phosphatase and tensin homolog) that dephosphorylates upstream IP3, the product of PI3K, which plays a role in platelet activation and reduces the number of circulating platelets by attenuating megakaryopoiesis (Weng et al., 2010; Kauskot et al., 2013). An increased expression of PTEN through statins might be operable either by an enhanced translation in the platelet or reduced suppression in megakaryocytes by NfkappaB. Simvastatin inhibits the binding of NfkappaB to the recognition sites NRF1/2 in the PTEN promotor (Ghosh-Choudhury et al., 2010). Moreover, statins inhibit even at low concentrations *in vitro* NfkappaB by activation of ERK5 (extracellular-signal-regulated kinase 5=MAP3Kinase7), which is well-expressed in platelets (Burkhart et al., 2012; Wu et al., 2013).

Taken together, a variety of studies demonstrated direct or indirect effects of statins on platelet activation, subsequently leading to a reduced expression and secretion of pro-inflammatory chemokines, cytokines as well as inflammatory molecules such as P-selectin and soluble CD40 ligand on this way affecting atherosclerotic lesion formation. For the clinician and the decision making the way statins exert their beneficial effects in cardiovascular patients may be of secondary importance. As a tool to understand why statins protect from atherosclerosis they aid in identifying multiple targets for future interventions. A limitation in the translation of *in vitro* studies to the clinical situation is that the observed effects of statins were mostly elicited at micromolar concentrations, more than orders of magnitude higher than what can be achieved even with high oral statin doses.

Maybe indeed the anti-platelet and other pleiotropic effects are of greater importance than estimated if we consider the missing evidence for cardiovascular survival of other cholesterol reducing agents such as ezetimibe and fibrates. Ezetimibe lowers LDL cholesterol by inhibiting the intestinal resorption and fibrates reduce LDL cholesterol by enhancing the catabolic activity via PPARα. The effect sizes of both agents on LDL are smaller compared with statins. Whether the antithrombotic and anti-inflammatory effects of statins are results of the inhibition of a concomitant off-target effect reducing isoprenoids might be tackled when PCSK9-inhibitors, a promising new class of strong LDL-reducing agents that do not affect the substrates of HMG-CoA, will be scrutinized.

## Conclusion

Our current assessment of the role of platelets for atherogensis is vastly driven by the insights gleaned from animal models of atherosclerosis that allow to study the role of single molecules in a cell and tissue specific manner by using conditional targeted genes. Commonly platelet specific expression or deletion is achieved using the CXCL4 promotor (PF4-Cre). The answers that can be obtained are as good as the models of atherosclerosis that are available, typically apoE- or LDLR- deficient mice put on cholesterol rich diet, which copy some but not all of the characteristics of human atherosclerosis. We have to acknowledge that our understanding of human atherogenesis is still very limited, which is in part due to the technical difficulty in assessing reliably, non-invasively and with reasonable efforts the atherosclerotic burden over time in humans leading to the employment of sometimes weak surrogate parameters such as Intima-Media thickness and soft end points. Hard endpoints, however, do not tell us whether beneficial effects by antiplatelet drugs on cardiovascular mortality, stroke and myocardial infarction derive from fewer plaque ruptures due to alterations in the size and stability of atherosclerotic lesions or rather from mere thrombotic and hemostatic inhibition. Examples are the oral GPIIb/IIIa antagonists that failed to keep their promises or the side effects of PAR-1 antagonists demonstrating the difficulty to separate clearly antithrombotic and anti-inflammatory properties from antihemostatic effects. An increase in understanding and dissecting the specific role of platelets in atherosclerosis compared to thrombosis has to be envisioned to result in novel anti-platelet targets and therapies, which are clearly needed to improve the prevention of atherosclerotic complications.

### Conflict of interest statement

Philipp von Hundelshausen is shareholder of Carolus Therapeutics Inc. The authors declare that the research was conducted in the absence of any commercial or financial relationships that could be construed as a potential conflict of interest.
